# Different physiologic biomechanical metrics correlate with aortic diameter increases in normal maturation compared to aneurysm progression in mice

**DOI:** 10.1016/j.jmbbm.2025.107105

**Published:** 2025-06-20

**Authors:** Yufan Wu, Krashn Kumar Dwivedi, Jacob Rother, Maya K. Sumra, Jessica E. Wagenseil

**Affiliations:** Department of Mechanical Engineering and Materials Science, Washington University in St. Louis, St. Louis, MO, United States

**Keywords:** Thoracic aortic aneurysm, Marfan syndrome, Elastin, Collagen, Biomechanics, Constitutive modeling

## Abstract

Thoracic aortic aneurysm (TAA) is the major cardiovascular manifestation of Marfan Syndrome (MFS), a connective tissue disorder caused by mutations in fibrillin-1. Aneurysmal dilation usually occurs in the ascending aorta (ASC) in MFS, but structural and mechanical changes are detectable throughout the arterial tree that may lead to dissection and rupture. Clinical management includes measuring ASC diameter and/or growth rate but does not typically include other regions of the thoracic aorta and dissection in the descending aorta (DSC) can occur after surgical replacement of the ASC. In severe forms of MFS, dilation forms concomitantly with aortic maturation, so it can be difficult to separate normal and pathologic changes in diameter. We used *Fbn1*^*mgR/mgR*^ (MU) (a model of severe MFS) and wildtype (WT) mice and quantified biaxial physiologic biomechanical metrics of ASC and DSC at 1, 2, 3, and 4 months of age, which includes a period of normal growth and aortic maturation in WT mice and aneurysm formation in MU mice. The results showed age- and location-specific dilation and alterations in biomechanical metrics with different patterns in WT and MU aorta. A multivariable mixed model showed that stored strain energy and circumferential stress were the primary contributors to aortic diameter predictions in WT ASC and DSC, while circumferential and axial incremental moduli (i.e. material stiffness) were the primary contributors to aortic diameter predictions in MU ASC and DSC. The results highlight different biomechanical metrics associated with aortic diameter increases in normal maturation compared to TAA progression in mice

## 1. Introduction

Thoracic aortic aneurysms (TAA) are characterized by progressive dilation of the aortic wall that may eventually lead to dissection (tearing) or rupture (failure) and increased risk of death. Up to 20 % of TAAs are linked to single-gene mutations, including those associated with Marfan Syndrome (MFS) (Milewicz et al., 2019). MFS is a connective tissue disorder caused by mutations in the gene that encodes the extracellular matrix (ECM) glycoprotein fibrillin-1. Fibrillin-1 forms microfibrils, which help assemble elastic fibers, supporting the elastic function of soft tissues including the aorta (Godwin et al., 2019). Mutations in fibrillin-1 are linked to fragmentation and loss of stability in elastic fibers, which affects the biomechanical properties of the aorta (Asano et al., 2022) and triggers progressive ECM remodeling that contributes to the formation of TAA in MFS (Pereira et al., 1997). Fibrillin-1 alterations in MFS also lead to dysregulated transforming growth factor beta (TGF-β) signaling that causes changes in smooth muscle cell phenotype and additional ECM remodeling contributing to TAA progression (Neptune et al., 2003).

The progression of TAA in MFS involves increases in the aortic diameter over time and can occur concurrently with normal growth and maturation. The extent and rate of aortic dilation varies in MFS with 35 % of individuals exhibiting aortic root dilation by age 5, 70 % by age 20, and 80 % by age 40 (Aburawi et al., 2007). As ECM remodeling and evolution of biomechanical properties occur throughout postnatal maturation in the aorta (Bendeck et al., 1991, 1994; Le et al., 2015; McLean et al., 2005; Murtada et al., 2021; Weiss et al., 2024), it is critical to compare and contrast the changes during normal maturation with those during TAA progression. Dilation in MFS typically starts at the aortic root and extends into the ascending aorta (ASC). Other parts of the aorta, including the descending (DSC), can show dilation that often manifests after surgical replacement of proximal aortic regions (Mimoun et al., 2011). Both the ASC and DSC show evidence of ECM remodeling and changes in wall mechanical metrics in MFS (Bellini et al., 2016; Cavinato et al., 2021; Dwivedi et al., 2025), but the ASC is more likely to develop an aneurysm than the DSC (Jimenez-Altayo et al., 2017; van Hout et al., 2022). As different parts of the aorta have different amounts and organization of ECM and wall biomechanical metrics (Bersi et al., 2017; Ferruzzi et al., 2015), it is important to determine how these baseline differences may contribute to variations in aneurysm susceptibility.

Studies that investigate the progression of TAA over time in MFS using mouse models usually focus on the aortic root and ASC (Cook et al., 2015; Habashi et al., 2006). Dwivedi et al. (2024) showed age- and sex-specific progression of TAA in the ASC for a severe MFS mouse model (*Fbn1*^*mgR/mgR*^ = MU) compared to wildtype (WT) controls and concluded that different timelines of TAA progression and survival rates between male and female mice may be related to the time course of changes in aortic wall geometry, ECM remodeling, and circumferential stiffness. These results in the severe MFS model were supported by those in a mild mouse model of MFS (*Fbn1*^*C1041G/+*^) that found aging-associated aortic stiffening exacerbated TAA progression (Gharraee et al., 2022). Studies that investigate differential TAA susceptibility in various aortic regions (such as the ASC and DSC) usually include only a single timepoint. Bellini et al. (2016) reported biaxial mechanical differences between ASC and DSC of MU and WT male mice at two months of age and concluded that increased circumferential material stiffness and loss of energy storage ability in ASC compared to DSC may contribute to aneurysm formation at this location. Dwivedi et al. (2025) showed that elastic fiber degradation and circumferential material stiffness correlated with aortic dilation in multiple aortic regions for MU mice at 4 months of age. Studies that investigate biaxial biomechanical metrics over time in multiple aortic regions may identify underlying mechanisms associated with normal aortic maturation compared to aneurysm progression and location-specific aneurysm susceptibility.

This study aimed to quantify the biaxial mechanical behavior of ASC and DSC in *Fbn1*^*mgR/mgR*^ (MU), a severe model of MFS, and wildtype (WT) mice over maturation and TAA progression from 1 to 4 months of age. Physiologic equivalent biomechanical metrics were obtained using biaxial inflation experiments and a two fiber family Holzapfel-Gasser-Ogden (HGO) constitutive model (Holzapfel et al., 2000). We used multiphoton microscopy to examine changes in the major ECM components of the aortic wall that may contribute to changes in biomechanical metrics. Multivariable linear regression was performed to determine the primary contributors for predicting the aortic diameter in WT and MU aorta. Our results show that different biomechanical metrics predict the diameter increases in normal maturation (WT), compared to aneurysm progression (MU). The circumferential and axial moduli (i.e. material stiffness), in particular, may be useful biomechanical biomarkers for predicting TAA dilation associated with MFS in the ASC and DSC.

## 2. Materials and methods

### 2.1. Mice

All protocols were approved by the Washington University Institutional Animal Care and Use Committee and conform to current NIH guidelines. Male and female *Fbn1*^*mgR/mgR*^ (MU), a severe model of MFS (Pereira et al., 1999), and wild-type (WT) littermate mice were used to collect passive biaxial mechanical data and ECM images of the ASC and DSC segments of thoracic aorta. The mechanical data and ECM images were collected at the ages of 1, 2, 3 and 4 months. The mice were euthanized by CO_2_ inhalation in a Smart Box and ASC and DSC thoracic aortic segments were isolated and stored in phosphate-buffered saline (PBS) at 4 °C. All mechanical tests were performed within three days of aortic removal (Amin et al., 2011).

### 2.2. Passive biaxial mechanical testing

Prior to mechanical testing, the aortic segments were brought to room temperature for at least 30 min. Ex vivo biaxial mechanical tests were performed as previously reported (Dwivedi et al., 2024; Kailash et al., 2024). Briefly, the ASC and DSC were submerged in PBS at 37 °C and mounted on custom stainless cannulae in a pressure myograph (110P, Danish Myotechnology) using 7.0 sutures. The unloaded length of the aortic segment was determined as the length at which there was no change in force by moving the micrometer ±10 μm. The in vivo axial stretch ratio was defined as the axial stretch ratio where there was almost no change in force when the aorta was inflated from 0 to 150 mmHg (Ferruzzi et al., 2013). After determining the in vivo stretch ratio, the aortic segment was preconditioned by cyclic inflation three times from 0 to 150 mmHg at the in vivo stretch ratio, followed by three cyclic axial stretches between the in vivo axial stretch ratio ± (0.1–0.2) at 150 mmHg. Six mechanical test protocols were then performed: three inflation protocols (0–150 mmHg) at three different fixed axial stretch ratios (0.1–0.2 below in vivo stretch ratio, in vivo stretch ratio, and 0.1–0.2 above in vivo stretch ratio) and three axial stretch protocols (from the lowest to highest axial stretch ratios) at three different fixed pressures (50, 100 and 150 mmHg). The rate of loading and unloading was 3 mmHg/s during the inflation cycles and 0.01–0.05 mm/s during the axial stretch cycles. The third loading cycle of each protocol was used for further data analysis. After mechanical testing, each aortic segment was cut into rings and imaged for unloaded dimension measurements. Data for a single inflation cycle for the ASC at all timepoints and for the ASC and DSC at 4 months of age were reported in previous publications (Dwivedi et al., 2024, 2025).

### 2.3. Data analysis for mechanical testing

The ASC and DSC were assumed to be fully incompressible with no shear under loading. The mid wall circumferential ($\lambda_{\theta }$), axial ($\lambda_{z}$), and radial ($\lambda_{r}$) stretch ratios were calculated by,

(1a) $$\lambda_{\theta }=\frac{1}{2}\left(\frac{d_{i}}{D_{i}}+\frac{d_{o}}{D_{o}}\right)$$

(1b) $$\lambda_{z}=\frac{l}{L}$$

(1c) $$\lambda_{r}=\frac{1}{\lambda_{\theta }\lambda_{z}}$$

where l and L are the loaded and unloaded lengths of the aortic segment, d and D are the loaded and unloaded diameters, and subscripts i and o represent the inner and outer surfaces, respectively.

The average experimental Cauchy stresses in the circumferential ($\sigma_{\theta }$) and axial directions ($\sigma_{z}$) were calculated by,

(2a) $$\sigma_{\theta }=\frac{Pd_{i}}{d_{o}-d_{i}},$$

(2b) $$\sigma_{z}=\frac{4f+P\pi d_{i}^{2}}{\pi (d_{o}^{2}-d_{i}^{2})}$$

where P and f are the measured pressure and axial force, respectively.

### 2.4. Constitutive modeling

The mechanical behavior of the ASC and DSC were modeled using the HGO strain energy density function (W) (Eqn. (3a)) that incorporates contributions of an isotropic, Neo-Hookean matrix ($W_{iso}$) (Eqn. (3b)) embedded with anisotropic, exponential fibers ($W_{ani}$) (Eqn. (3c)) (Holzapfel et al., 2000),

(3a) $$W=W_{iso}+W_{ani}$$

(3b) $$W_{iso}=\frac{c}{2}(I_{1}-3)$$

(3c) $$W_{ani}=\sum_{j=1}^{2}\frac{k_{1}}{2k_{2}}\left(e^{k_{2}{\left(I_{4}^{j}-1\right)}^{2}}-1\right)$$

where c>0 is an elastic modulus-like parameter, $I_{1}=\lambda_{\theta }^{2}+\lambda_{z}^{2}+\lambda_{r}^{2}$ is the first invariant, $k_{1}>0$ is an elastic modulus-like parameter, $k_{2}>0$ is a dimensionless parameter associated with material nonlinearity, $I_{4}^{j}=\lambda_{\theta }^{2}\;cos^{2}(\alpha^{j})+\lambda_{z}^{2}sin^{2}(\alpha^{j})$ is the fourth invariant and denotes the stretch of two symmetric fiber-families oriented at an angle α with respect to the circumferential direction, where $\alpha^{1}=+\alpha$; $\alpha^{2}=-\alpha$.

The stresses predicted from the strain energy function for an incompressible cylinder can be calculated as,

(4) $$\sigma =2F\frac{\partial W}{\partial C}F^{T}-pI$$

where $F=diag[\lambda_{\theta },\lambda_{z},\lambda_{r}]$ (no shear), $C=diag[\lambda_{\theta }^{2},\lambda_{z}^{2},\lambda_{r}^{2}]$ (no shear), p is the Lagrange multiplier, and I is the identity tensor. Best fit parameters were obtained from constrained nonlinear regression in Matlab (Mathworks), using the fmincon function. The error function to be minimized was,

(5) $$error=\sqrt{\frac{\sum {\left(\sigma_{\theta ,exp}(i)-\sigma_{\theta ,pred}(i)\right)}^{2}}{\sum {\left(\sigma_{\theta ,exp}(i)\right)}^{2}}}+\sqrt{\frac{\sum {\left(\sigma_{z,exp}(i)-\sigma_{z,pred}(i)\right)}^{2}}{\sum {\left(\sigma_{z,exp}(i)\right)}^{2}}}$$

A penalty function was imposed if the predicted circumferential stress from the isotropic material did not contribute to more than 10 % of the overall circumferential stress (Cheng et al., 2013). With the penalty function, the overall error function was,

(6a) $$error\;new=error+\Sigma ({\text{penalty}(i)}^{2})$$

(6b) $$penalty(i)=0.1-\frac{\sigma_{\theta ,pred,iso}}{\sigma_{\theta ,pred,total}}\;if\;\frac{\sigma_{\theta ,pred,iso}}{\sigma_{\theta ,pred,total}}<0.1$$

The circumferential and axial moduli were derived from the strain energy function and applied stretch ratios as measures of material stiffness (Baek et al., 2007),

(7a) $$\zeta_{\theta }=4\lambda_{\theta }^{2}\frac{\partial W}{\partial C_{\theta \theta }}+4\lambda_{\theta }^{4}\frac{\partial^{2}W}{\partial C_{\theta \theta }^{2}},$$

(7b) $$\zeta_{z}=4\lambda_{z}^{2}\frac{\partial W}{\partial C_{zz}}+4\lambda_{z}^{4}\frac{\partial^{2}W}{\partial C_{zz}^{2}}$$

The average value of the mean blood pressure for each genotype and age group from our previous study (Table 1) (Dwivedi et al., 2024) was used for calculating physiologic biomechanical metrics (i.e. $\lambda_{\theta },\lambda_{z},W,\sigma_{\theta },\sigma_{z},\zeta_{\theta },\zeta_{z},$).

### 2.5. Multiphoton imaging

Multiphoton imaging was performed on aortic rings as described in our previous study (Dwivedi et al., 2024). Briefly, the aortic rings after mechanical testing were fixed in 4 % paraformaldehyde overnight, washed 3 times in PBS, sequentially dehydrated in increasing concentrations of ethanol (30 %, 50 %, and 70 %), and stored in 70 % ethanol at 4 °C for imaging of the wall structure. ASC and DSC rings were cryosectioned in 20 μm thick sections, mounted on slides, and stained with DAPI for cell nuclei. Z-stacks were collected using an sp-8 DIVE multiphoton confocal microscope (Leica) with a 60× objective and 2 μm step size. Collagen fibers, elastic fibers, and cell nuclei were excited at 880 nm and imaged using second harmonic generation (SHG) (emission, 420–460 nm), autofluorescence (emission, 495–540 nm), and DAPI (emission, 430–450 nm), respectively.

### 2.6. Statistical analysis

Samples with fitted material constants identified as outliers were excluded from all analyses. Outliers were identified using Matlab isoutlier function and defined as >3 standard deviations from the mean. 3 samples out of 118 and 3 samples out of 124 were identified as outliers for the ASC and DSC, respectively. We had four independent variables to consider: sex, age, vessel, and genotype. Our previous work showed a significant, but relatively small effect size for sex compared to age, vessel, and genotype (Dwivedi et al., 2024, 2025). As the number of samples per group was not powered to investigate small effect sizes, we combined data from male and female mice. Further statistical analysis was performed in Prism (GraphPad). Experimental groups were compared using three-way ANOVA to assess the effects of the independent variables age (A), vessel (V), and genotype (G), and interactions between the independent variables on the biomechanical metrics. P < 0.05 was considered significant.

Mixed multiple regression models in R Studio were constructed using forward stepwise procedures. For each biomechanical metric, data from all samples were combined into a single column, and Z-stack normalization was performed. The best mixed model for predicting the physiologic deformed inner diameter was chosen with P < 0.05 and variance inflation factor (VIF) < 3 to ensure significant correlation and preclude strong collinearity between biomechanical metrics, respectively. When two biomechanical metrics were determined to be correlated (VIF ≥3), the metric with the highest relative contribution to the predicted deformed inner diameter was retained in the model, and the other metric was excluded.

## 3. Results

### 3.1. Undeformed diameter, physiologic stretch ratios, and deformed diameter

**Fig. 1.**
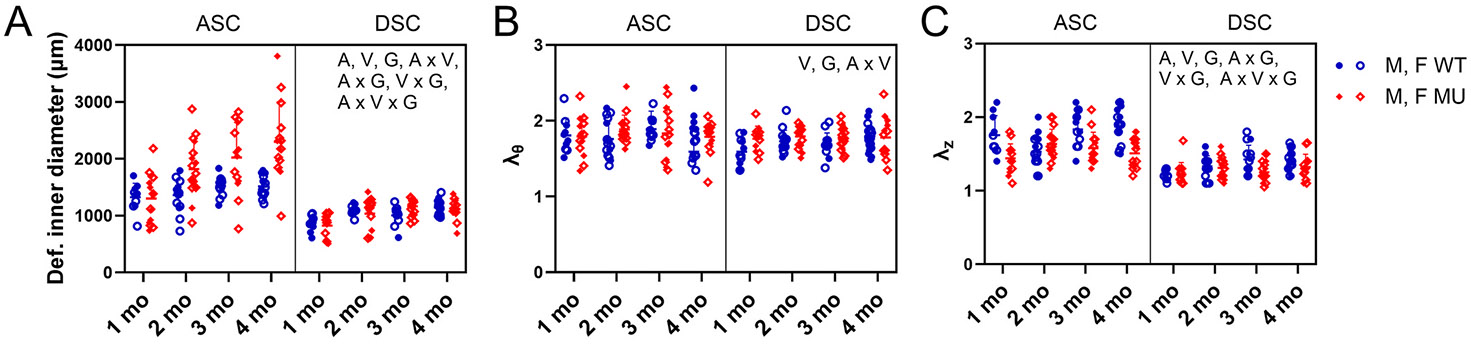
Deformed inner diameter (A), circumferential stretch ratio ($\lambda_{\theta }$) (B), and axial stretch ratio ($\lambda_{z}$) (C) for each group. Letters indicate significant effects by 3-way ANOVA for the independent variables age (A), vessel (V), and genotype (G) and all interactions. Males and females are plotted with different symbols but are not separated by sex for statistical analyses. Individual data points and mean ± SD are shown. N = 10–19/group.

The undeformed outer and inner diameters were measured from cut rings after mechanical testing (Supp. Fig. S1). For the undeformed outer and inner diameters, all independent variables (age, vessel, and genotype) and all possible interactions between independent variables played a significant role. The deformed inner diameter (Fig. 1A) was calculated from the pressure-diameter data at the in vivo stretch ratio for each group’s average mean blood pressure (Table 1). For the deformed inner diameter, age (p < 0.0001), vessel (p < 0.0001), genotype (p < 0.0001), interactions between age and vessel (p = 0.0482), age and genotype (p = 0.0194), vessel and genotype (p < 0.0001), and age, vessel, and genotype (p = 0.0194) were significant (Fig. 1A). Across all ages, MU inner diameter was 30 % and 1 % higher than WT in ASC and DSC, respectively. Across all ages, ASC inner diameter was 39 % and 80 % higher than DSC in WT and MU, respectively. Inner diameter increased with age in WT and MU ASC and DSC but increased the most in MU ASC. For the physiologic circumferential stretch ratio, vessel (p = 0.0372), genotype (p = 0.0082), and the interaction between age and vessel (p = 0.0314) were significant (Fig. 1B). Across all ages, the circumferential stretch ratio was 4 % higher in MU compared to WT in ASC and 5 % in DSC. Across all ages, the circumferential stretch ratio was 4 % higher in ASC compared to DSC in WT, and 3 % in MU. For the physiologic axial stretch ratio, age (p = 0.0021), vessel (p < 0.0001), genotype (p < 0.0001), interaction between age and genotype (p < 0.0001), interaction between vessel and genotype (p = 0.0078), and interaction between age, vessel, and genotype (p = 0.0057) were significant (Fig. 1C). Across all ages, the axial stretch ratio was 12 % and 5 % lower in MU compared to WT ASC and DSC, respectively. Across all ages, the axial stretch ratio was 23 % and 17 % higher in ASC compared to DSC WT and MU, respectively. The axial stretch ratio typically increased with age. A summary of the significance and percent total variation from the three-way ANOVA for each independent variable and their interactions is presented in Supplemental Table S1.

Overall, diameter increases from 1 to 4 months of age in the mouse were observed in both WT and MU ASC and DSC, but the rate of diameter increase for MU ASC was higher than all other groups, demonstrating pathological increases associated with TAA that were more extensive than aortic normal maturation. There were mild differences in the circumferential and axial stretch ratios due to vessel and genotype. The axial stretch ratio, and not the circumferential stretch ratio, showed dependence on age and interactions between genotype and age, indicating that the axial stretch ratio showed complex adaptation for aortic maturation and disease over time.

### 3.2. Material parameters

**Fig. 2.**
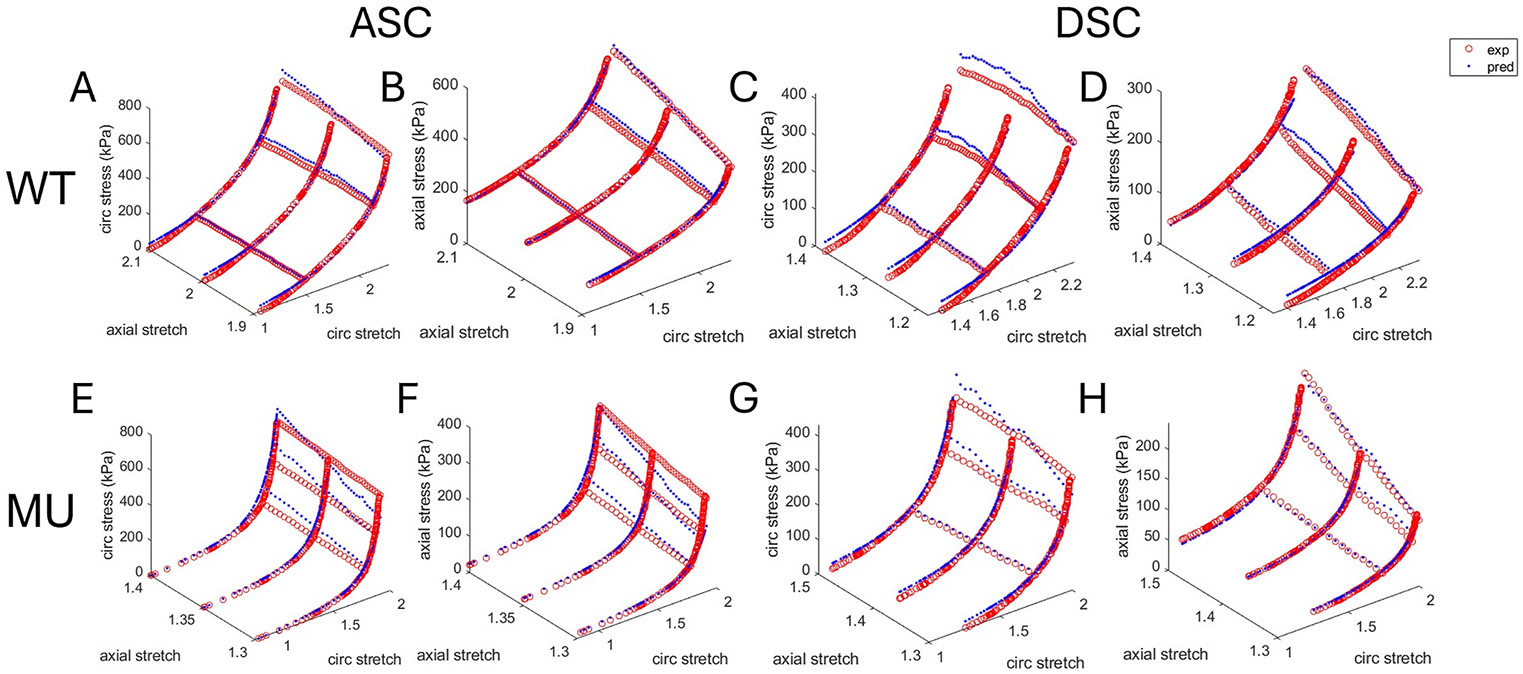
Representative experimental (exp) and HGO-model predicted (pred) stretch-stress data for 4-month-old WT (A–D) and MU (E–H) ASC (A, B, E, F) and DSC (C, D, G, H). Representative data for ages 1, 2, and 3 months are in the supplemental figures.

**Fig. 3.**
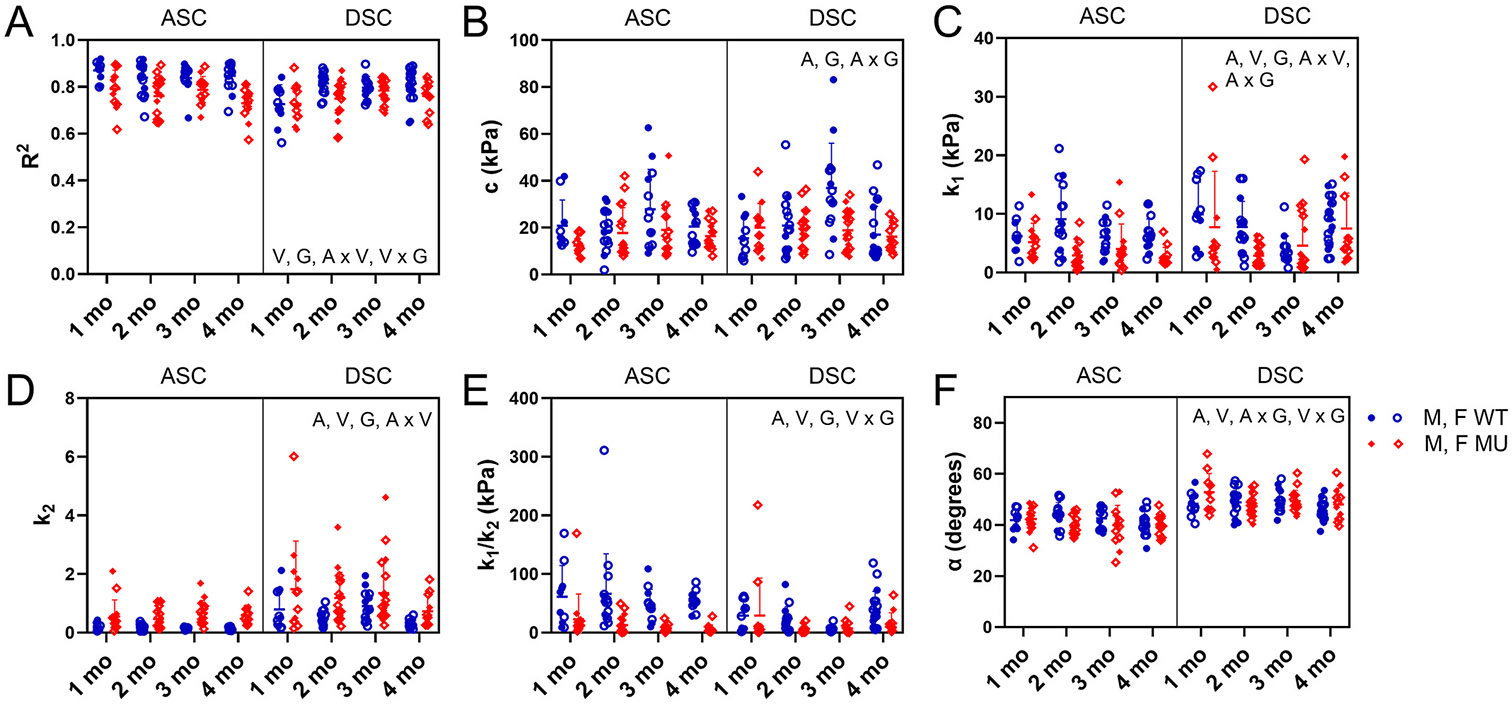
Fitting performance and fitted material parameters for the HGO constitutive model (Eqn. (3)). The fitting performance is demonstrated by the R^2^ value (A). The elastic modulus-like parameter of the isotropic, Neo-Hookean material is c (B). The elastic modulus-like parameter of the anisotropic, nonlinear material is $k_{1}$ (C). The exponential constant of the anisotropic, nonlinear material is $k_{2}$ (D). The ratio, $k_{1}∕k_{2}$, represents an inverse measure of the degree of nonlinearity of the anisotropic, nonlinear material (E). The angle, α, is the orientation of the fiber-families in the nonlinear, anisotropic material with respect to the circumferential direction (F). Letters indicate significant effects by 3-way ANOVA for the independent variables age (A), vessel (V), and genotype (G) and all interactions. Males and females are plotted with different symbols but are not separated by sex for statistical analyses. Individual data points and mean ± SD are shown. N = 10–19/group.

The HGO constitutive model had reasonable fitting performance for WT and MU ASC and DSC, as shown by the representative examples of experimental and model predicted stretch-stress behavior in Fig. 2 for 4 month old mice. Representative fits for ages 1, 2, and 3 months are shown in Supplemental Figures S2 (ASC) and S3 (DSC). Fig. 3 shows the goodness of fit, *R*^*2*^, and the fitted material parameters for the HGO constitutive model for each group. For *R*^*2*^, vessel (p < 0.0001), genotype (p < 0.0001), interaction between age and vessel (p = 0.0007), and interaction between vessel and genotype (p = 0.0067) were significant (Fig. 3A). *R*^*2*^ for MU had more variation within groups than WT. Across all ages, *R*^*2*^ was 10 % and 4 % lower in MU than WT for ASC and DSC, respectively, and 4 % lower in DSC than ASC overall. For c, the elastic modulus-like parameter of the isotropic, Neo-Hookean material, age (p < 0.0001), genotype (p = 0.0010), and interaction between age and genotype (p = 0.0063) were significant (Fig. 3B). c typically increased with age. Across all ages, c was 26 % lower and 17 % lower in MU than WT ASC and DSC, respectively. For $k_{1}$, the elastic modulus-like parameter of the anisotropic, nonlinear material, age (p < 0.0266), vessel (p = 0.0245), genotype (p < 0.0001), interaction of age and vessel (p = 0.0072), and interaction of age and genotype (p = 0.0107) were significant (Fig. 3C). $k_{1}$ decreased with age in ASC and showed no specific trend with age in DSC. Across all ages, MU $k_{1}$ was 48 % and 24 % lower than WT in ASC and DSC, respectively. For $k_{2}$, the parameter that describes the degree of nonlinearity of the anisotropic, nonlinear material, age (p = 0.0163), vessel (p < 0.0001), genotype (p < 0.0001), and interaction between age and vessel (p = 0.0418) were significant. $k_{2}$ typically increased with age. Across all ages, MU had 277 % and 94 % higher $k_{2}$ than WT ASC and DSC, respectively (Fig. 3D). Across all ages, DSC had 303 % and 107 % higher $k_{2}$ than ASC in WT and MU, respectively. For the ratio $k_{1}∕k_{2}$, which is inversely related to the nonlinearity of the stretch-stress behavior, age (p = 0.0271), vessel (p < 0.0003), genotype (p < 0.0001), and interaction of vessel and genotype (p < 0.0001) were significant (Fig. 3E). $k_{1}∕k_{2}$ typically decreased with age. Across all ages, MU had 78 % and 42 % lower $k_{1}∕k_{2}$ than WT in ASC and DSC, respectively. DSC had 57 % lower and 14 % higher $k_{1}∕k_{2}$ than ASC in WT and MU, respectively. For the fiber alignment angle, α, age (p = 0.0282), vessel (p < 0.0001), interaction of age and genotype (p = 0.0139), and interaction of vessel and genotype (p = 0.0160) were significant (Fig. 3F). α typically decreased with age. Across all ages, DSC had 14 % and 20 % higher α than ASC in WT and MU, respectively, indicating a more axial orientation of the symmetric fiber-families.

Overall, reduced goodness of fit (*R*^*2*^), lower isotropic constant (c), and higher nonlinearity parameters ($k_{2}$, inverse of $k_{1}∕k_{2}$) were seen in MU ASC and DSC compared to WT and in DSC MU and WT compared to ASC.

### 3.3. Physiologic biomechanical metrics

#### 3.3.1. Stored strain energy

**Fig. 4.**
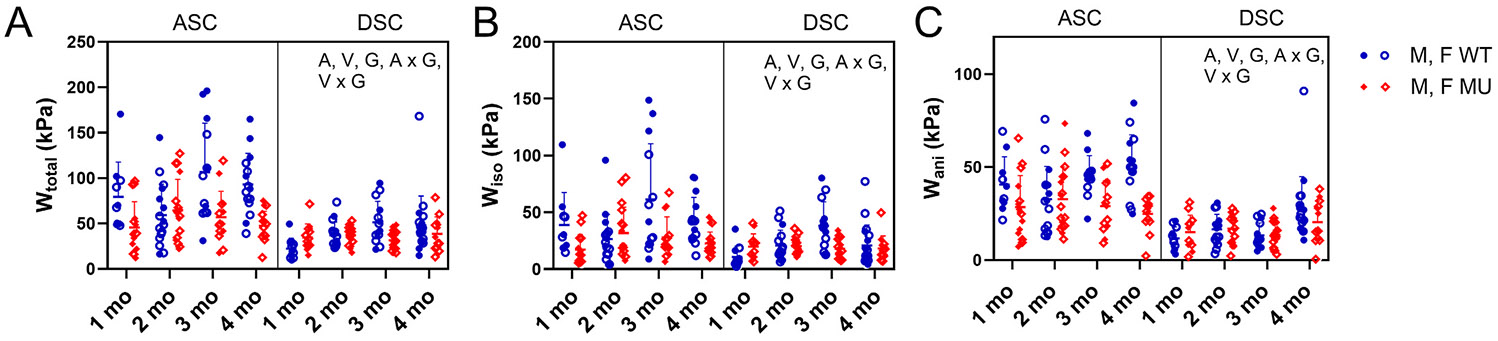
The stored strain energy (W) for the total composite material (A) and the contributions of the isotropic, Neo-Hookean ($W_{iso}$) (B) and anisotropic, nonlinear ($W_{ani}$) (C) materials (Eqn. (3)) . Letters indicate significant effects by 3-way ANOVA for the independent variables age (A), vessel (V), and genotype (G) and all interactions. Males and females are plotted with different symbols but are not separated by sex for statistical analyses. Individual data points and mean ± SD are shown. N = 10–19/group.

Fig. 4 shows the total strain energy (Fig. 4A) and the strain energy contributed by the isotropic, Neo-Hookean material (Fig. 4B) or the anisotropic, nonlinear material (Fig. 4C). For the total strain energy, age (p = 0.0118), vessel (p < 0.0001), genotype (p < 0.0001), interaction between age and genotype (p = 0.0008), and interaction between vessel and genotype (p = 0.0003) were significant (Fig. 4A). Across all ages, ASC stored 53 % and 33 % more total strain energy than DSC in WT and MU, respectively. MU stored 37 % and 11 % less total strain energy than WT ASC and DSC, respectively. Strain energy increased until about 3 months of age, then decreased or remained constant. For the strain energy contributed by the isotropic, Neo-Hookean material, age (p = 0.0009), vessel (p < 0.0001), genotype (p < 0.0001), interaction between age and genotype (p = 0.0008), and interaction between vessel and genotype (p = 0.0088) were significant (Fig. 4B). Consistent with the total strain energy, the strain energy contributed by the isotropic material increased up until 3 months of age, then decreased or remained constant. Across all ages, the strain energy contributed by the isotropic material was 41 % and 15 % higher in WT than MU in ASC and DSC, respectively. For the strain energy contributed by the anisotropic nonlinear material, age (p = 0.0142), vessel (p < 0.0001), genotype (p < 0.0001), interaction between age and genotype (p = 0.0034), and interaction between vessel and genotype (p = 0.0002) were significant (Fig. 4C). The strain energy contributed by the anisotropic material increased throughout the time period included in the study. Across all ages, the strain energy contributed by the anisotropic material was 32 % and 5 % lower in MU compared to WT ASC and DSC and 59 % and 42 % higher in ASC compared to DSC WT and MU, respectively.

In general, MU ASC and DSC were less capable of storing strain energy compared to their WT counterparts, especially at higher ages. Overall, ASC stored more strain energy than DSC and stored strain energy increased with age.

#### 3.3.2. Average wall stresses

**Fig. 5.**
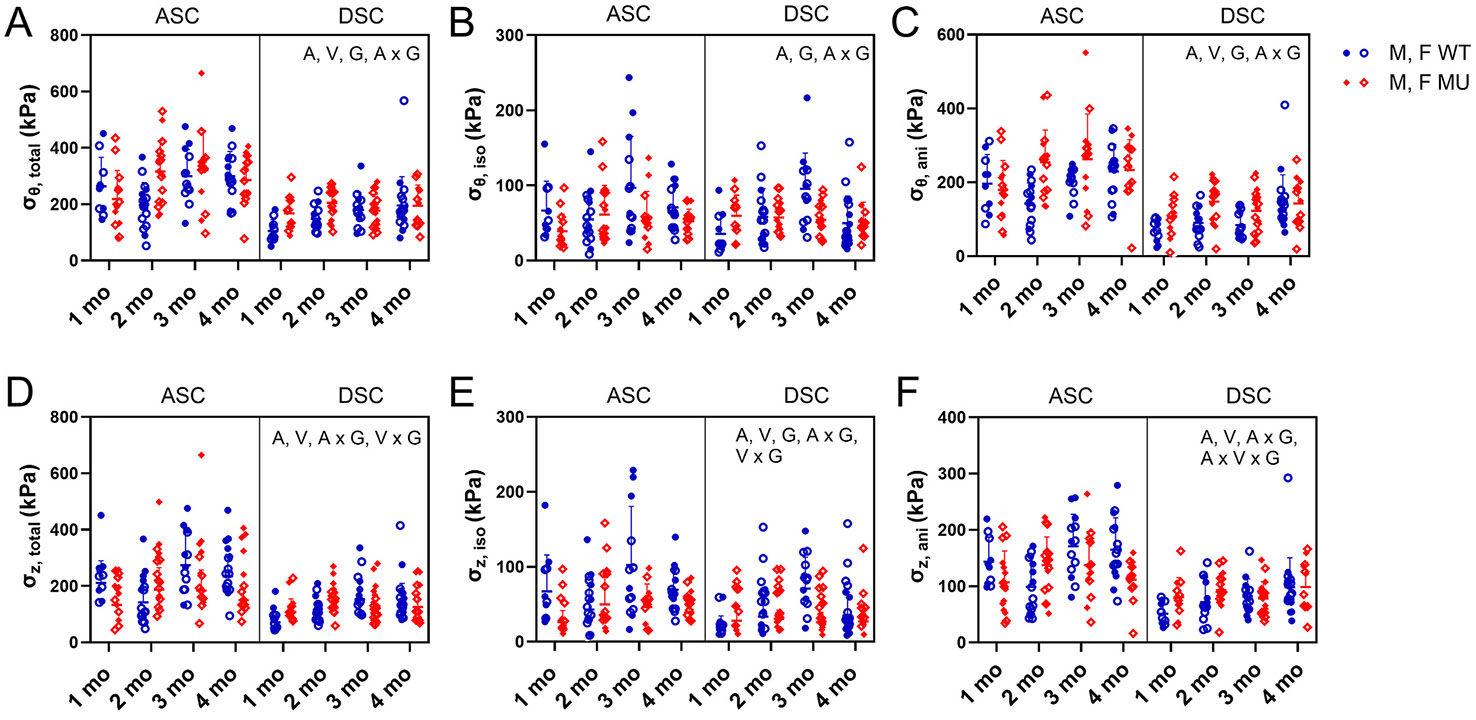
The circumferential ($\sigma_{\theta }$) (A–C) and axial stresses ($\sigma_{z}$) (D–F) calculated from Eqn. (4). The stresses are broken down into the total (A, D), contributions of the isotropic, Neo-Hookean (iso) (B, E) and anisotropic, nonlinear (aniso) (C, F) materials. Letters indicate significant effects by 3-way ANOVA for the independent variables age (A), vessel (V), and genotype (G) and all interactions. Males and females are plotted with different symbols but are not separated by sex for statistical analyses. Individual data points and mean ± SD are shown. N = 10–19/group.

Fig. 5 shows the predicted circumferential and axial stresses for the total composite and the individual contributions from the isotropic, Neo-Hookean and anisotropic, nonlinear materials. For the total predicted circumferential stress, age (p = 0.0021), vessel (p < 0.0001), genotype (p = 0.0310), and interaction between age and genotype (p = 0.0079) were significant (Fig. 5A). Total circumferential stress increased with age. Across all ages, total circumferential stress was 8 % and 18 % higher in MU than WT in ASC and DSC, respectively. Across all ages, total circumferential stress in ASC was 41 % and 35 % higher than DSC in WT and MU, respectively. For the total predicted axial stress, age (p = 0.0006), vessel (p < 0.0001), genotype (p = 0.0033), interaction between age and genotype (p = 0.0002), interaction between vessel and genotype (p = 0.0033), interaction between age, vessel, and genotype (p = 0.0253) were significant (Fig. 5D). Across all ages, total axial stress in MU was 24 % and 3 % lower for MU than WT in ASC and DSC, respectively. Across all ages, total axial stress in ASC was 45 % and 29 % higher than DSC in WT and MU, respectively.

The circumferential stress contributed by the isotropic, Neo-Hookean material significantly depended on age (p = 0.0007), genotype (p = 0.0107), and interaction between age and genotype (p = 0.0040) (Fig. 5B). Across all ages, the circumferential stress contributed by the isotropic material was 27 % lower in MU than WT for ASC and 7 % lower in DSC. The axial stress contributed by the isotropic Neo-Hookean material significantly depended on age (p < 0.0001), vessel (p < 0.0001), genotype (p < 0.0001), interaction between age and genotype (p < 0.0001), and interaction between vessel and genotype (p = 0.0309) (Fig. 5E). Across all ages, the axial stress contributed by the isotropic material was 43 % and 30 % lower in MU compared to WT ASC and DSC, respectively, and 44 % and 31 % higher in ASC compared to DSC WT and MU, respectively.

The circumferential stress contributed by the anisotropic, nonlinear material significantly depended on age (p = 0.0031), vessel (p < 0.0001), genotype (p < 0.0001), and interaction between age and genotype (p = 0.0027) (Fig. 5C). Across all ages, the circumferential stress contributed by the anisotropic material was 20 % and 34 % higher in MU compared to WT ASC and DSC, respectively, and 49 % and 44 % higher in ASC compared to DSC WT and MU, respectively. The axial stress contributed by the anisotropic nonlinear material significantly depended on age (p = 0.0017), vessel (p < 0.0001), interaction between age and genotype (p = 0.0019), interaction between vessel and genotype (p = 0.0051), and interaction between age, vessel, and genotype (p = 0.0495) (Fig. 5F). Across all ages, the axial stress contributed by the anisotropic material was 64 % and 46 % higher in ASC compared to DSC WT and MU, respectively.

In summary, ASC had higher circumferential stress than DSC, MU had higher circumferential stress than WT, especially for the contribution of the anisotropic material, and there was a decrease in axial stress in MU compared to WT at higher ages. The total circumferential and axial stresses increased with age.

#### 3.3.3. Incremental moduli

**Fig. 6.**
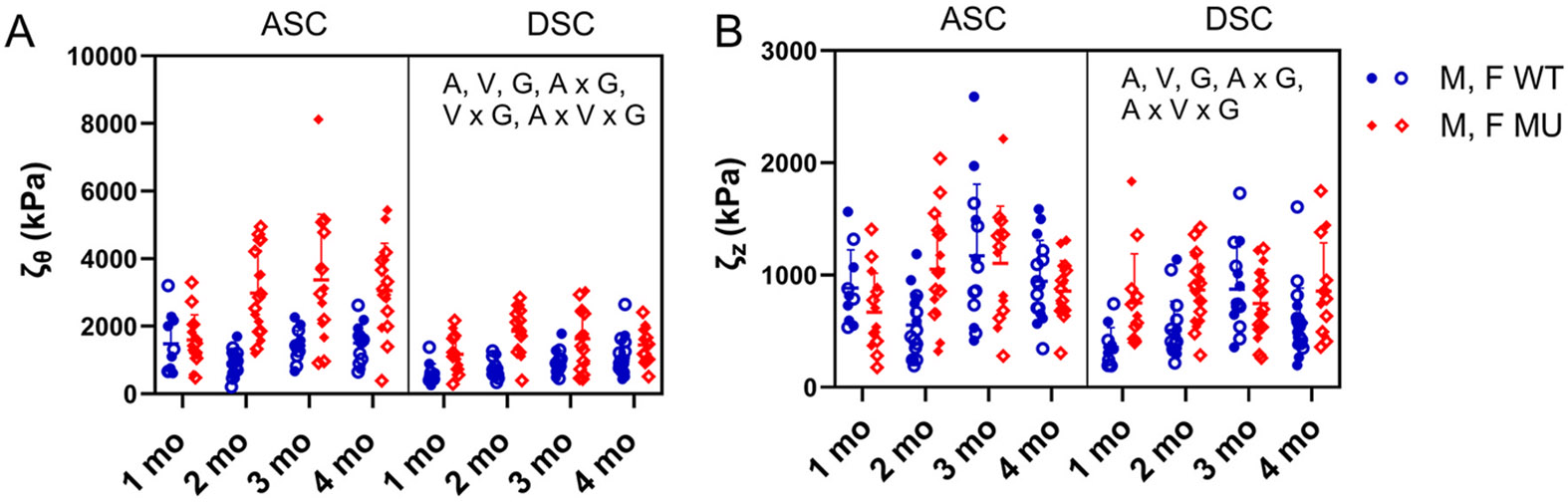
The circumferential ($\zeta_{\theta }$) (A) and axial ($\zeta_{z}$) (B) moduli (material stiffnesses) calculated from Eqn. (7). Letters indicate significant effects by 3-way ANOVA for the independent variables age (A), vessel (V), and genotype (G) and all interactions. Males and females are plotted with different symbols but are not separated by sex for statistical analyses. Individual data points and mean ± SD are shown. N = 10–19/group.

The incremental moduli (material stiffness) in the circumferential and axial directions are shown in Fig. 6. For the incremental modulus in the circumferential direction, age (p = 0.0010), vessel (p < 0.0001), genotype (p < 0.0001), interaction between age and genotype (p = 0.0014), interaction between vessel and genotype (p = 0.0016), and interaction between age, vessel, and genotype (p = 0.0415) were significant (Fig. 6A). The circumferential incremental modulus increased with age. The increase with age was most noticeable in the MU ASC, while the WT ASC and DSC showed only slight increases with age, and the MU DSC plateaued between 2 and 4 months of age. Across all ages, the circumferential incremental modulus was 106 % and 84 % higher in MU compared to WT in ASC and DSC, respectively. Across all ages, the circumferential incremental modulus was 38 % and 45 % higher in ASC compared to DSC in WT and MU, respectively. For the incremental modulus in the axial direction, age (p = 0.0003), vessel (p < 0.0001), genotype (p = 0.0097), interaction between age and genotype (p = 0.0013), and interaction between age, vessel, and genotype (p = 0.0287) were significant (Fig. 6B). The axial incremental modulus increased up to 3 months of age and then decreased or remained constant. The axial incremental modulus was 4 % and 43 % higher in MU compared to WT in ASC and DSC, respectively. Across all ages, the axial incremental modulus was 35 % and 13 % higher in ASC compared to DSC in WT and MU, respectively. In adult (3–4 month old) WT ASC and DSC, the circumferential and axial moduli had similar values, however in adult MU ASC and DSC, the circumferential modulus was 2–4 times higher than the axial modulus.

In short, progressive increases in circumferential modulus were observed in both ASC and DSC, with that of ASC increasing at a faster rate, and that of the MU ASC increasing at the fastest rate.

### 3.4. Mixed model correlation analyses

**Fig. 7.**
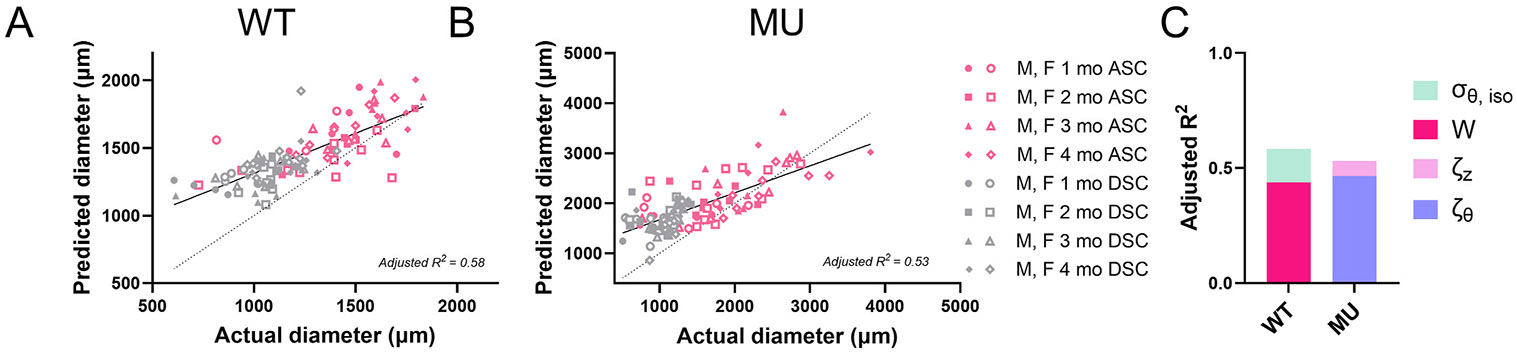
The best mixed model results predicting the deformed inner diameter for WT (A) and MU (B) aorta from the physiologic biomechanical parameters (C). $\zeta_{\theta }$ = circumferential modulus; W = total stored strain energy; $\sigma_{\theta ,iso}$ = circumferential stress contributed by the isotropic, Neo-Hookean material; $\zeta_{z}$ = axial modulus. The best fit line (solid), identity line (dotted), and adjusted R^2^ are shown in panels A and B. The relative contributions of each biomechanical parameter to the adjusted R^2^ are shown in panel C. The p-value, coefficients, and VIF are provided in Table 2.

We combined ASC and DSC data and ran separate mixed models for WT and MU mice to examine relationships between biomechanical metrics and physiologic deformed inner aortic diameter in normal maturation and aneurysmal dilation. The best mixed models for predicting the physiologic deformed inner diameter from biomechanical metrics for WT and MU aorta are shown in Fig. 7. The coefficients, p values, and VIF values are shown in Table 2. The mixed model results showed that for WT, the total strain energy and the circumferential stress contributed by the isotropic material were the main predictors of deformed inner diameter (Fig. 7A-C), with an adjusted R^2^ of 0.58. In contrast, for MU, the circumferential and axial incremental moduli were the main predictors of deformed inner diameter (Fig. 7B and C), with an adjusted R^2^ of 0.53. The results show that different biomechanical metrics are associated with aortic diameter increases during normal growth and maturation (WT) compared to aneurysmal dilation (MU).

We also combined MU and WT biomechanical metrics and ran separate mixed models for ASC and DSC to identify similarities and differences between aortic segments (Supplemental Fig. S4, Supp. Table 1). We found that circumferential modulus was the main predictor of deformed inner diameter in both ASC and DSC, although the secondary factor was different for each segment (circumferential stress for ASC and stored strain energy contributed by the anisotropic material for DSC). Additionally, DSC had a lower adjusted R^2^ (0.14) than ASC (0.46) due to the limited change in diameter overall with age and genotype in this aortic segment.

### 3.5. Representative multiphoton images of wall structure

**Fig. 8.**
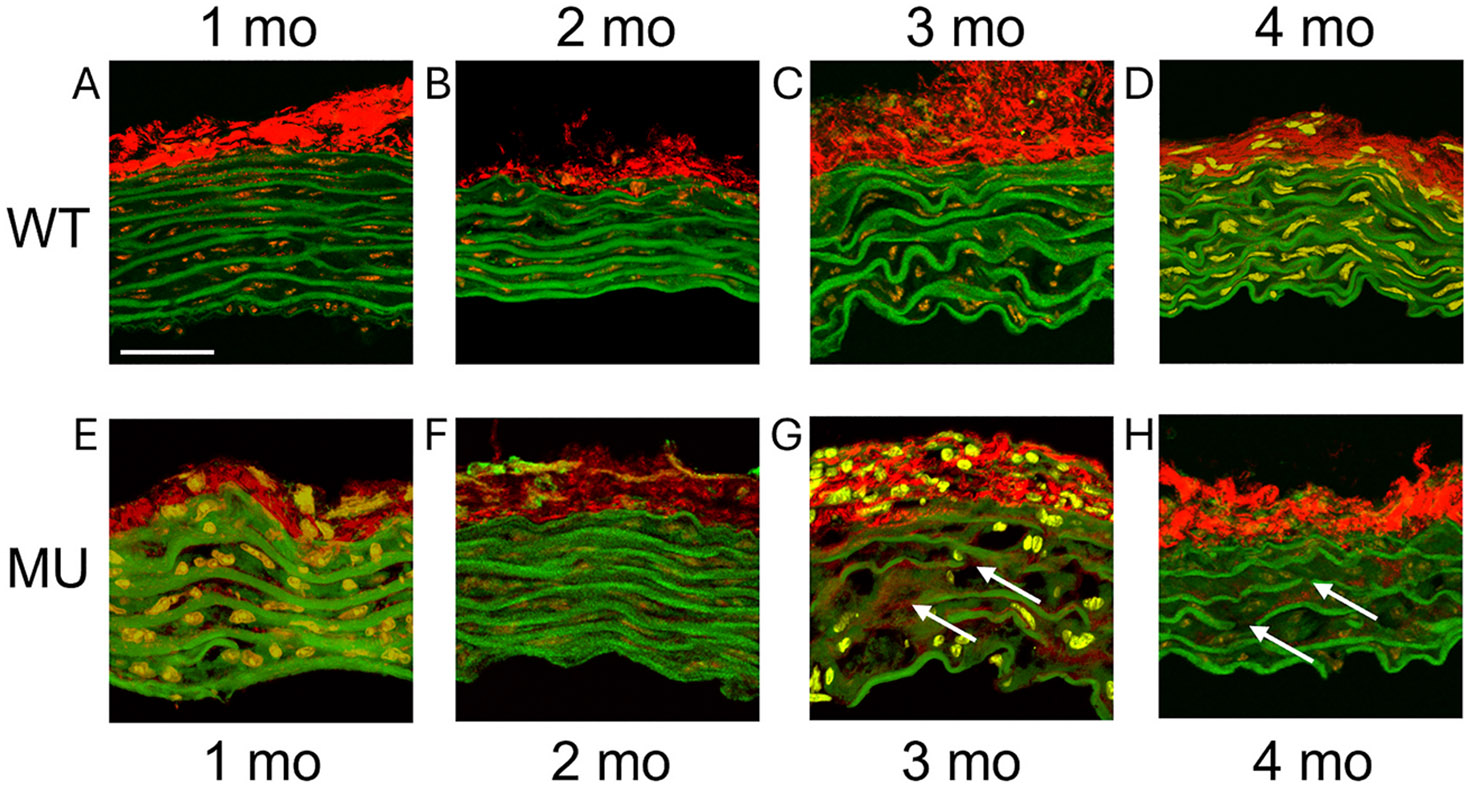
Representative multiphoton images of ASC cross-sections for WT (A–D) and MU (E–H) male mice at 1 (A, E), 2 (B, F), 3 (C, G), and 4 (D, H) months of age. Elastic fibers are shown in green, collagen fibers are shown in red, and cell nuclei are shown in orange/yellow. The lumen is at the bottom. Arrows (white) highlight elastic fiber fragmentation in 3 and 4 month old MU ASC. Scale bar = 25 μm.

**Fig. 9.**
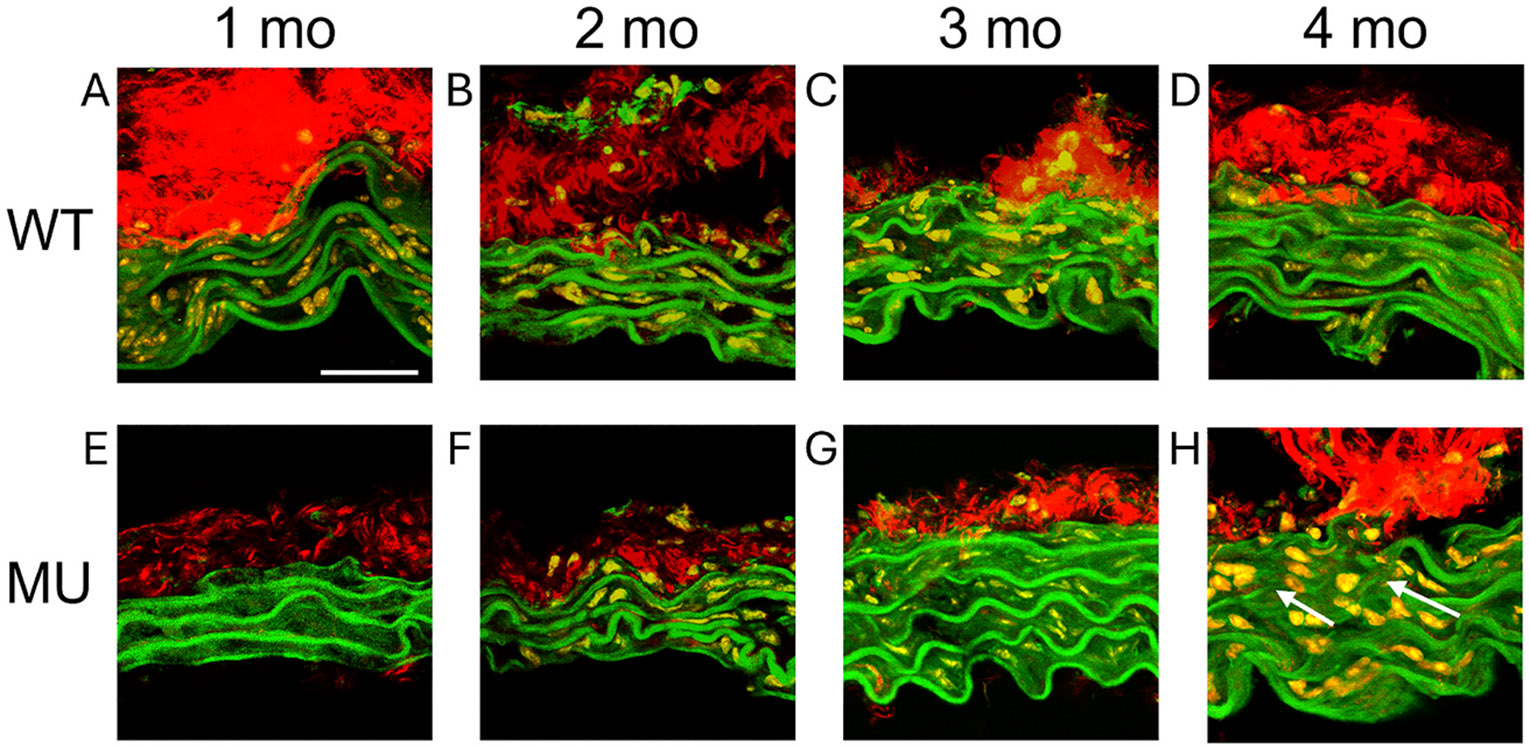
Representative multiphoton images of DSC cross-sections for WT (A–D) and MU (E–H) female mice at 1 (A, E), 2 (B, F), 3 (C, G), and 4 (D, H) months of age. Elastic fibers are shown in green, collagen fibers are shown in red, and cell nuclei are shown in orange/yellow. The lumen is at the bottom. Arrows (white) highlight elastic fiber fragmentation in 4 month old MU DSC. Scale bar = 25 μm.

Figs. 8 and 9 show the wall structure of WT and MU mice at ages 1, 2, 3, and 4 months for male ASC and female DSC, respectively. Elastic fiber fragmentation is visible in MU compared to WT in the ASC at 3 and 4 months of age and in the DSC at 4 months of age.

## 4. Discussion

We measured passive biaxial mechanics and examined the wall structure in ASC and DSC of a mouse model of severe MFS (MU) and littermate controls (WT) throughout normal maturation and aneurysm progression from 1 to 4 months of age. We fitted an HGO constitutive model to passive biaxial mechanical data and calculated physiologic biomechanical metrics based on the average mean blood pressure for each group to better understand how changes in biomechanical metrics with age, vessel, and genotype may be related to normal maturation and aneurysm progression. We used a multivariable mixed model to identify biomechanical metrics in WT and MU aorta that predicted the deformed inner diameter and may serve as biomarkers for more comprehensive clinical management of TAA.

### 4.1. HGO goodness of fit and interpretation of material parameters

The HGO constitutive model has been used extensively to describe the mechanical behavior of human and animal aorta (Holzapfel et al., 2000). We found that the goodness of fit (*R*^*2*^) for the HGO model was higher for WT than MU, and higher for ASC than DSC. A lower *R*^*2*^ can be caused by alterations to the ECM that significantly change the biaxial mechanical behavior of the aortic wall. For example, elastase-treated ASC with degraded elastic fibers had a much lower *R*^*2*^ for the HGO constitutive model than controls (Crandall et al., 2022). Our data and others (Cavinato et al., 2021; Dwivedi et al., 2024) showed progressive elastic fiber degradation and collagen fiber deposition in MU ASC that might lower the *R*^*2*^. Coupling of the fiber contributions in different directions can also affect the *R*^*2*^ of HGO-type models (Dong et al., 2021). Altered coupling of the fiber contributions in different arteries (Kailash et al., 2024) (i.e. DSC compared to ASC) or due to ECM remodeling in MU samples may also lower the *R*^*2*^.

The HGO constitutive model separates stress contributions into a Neo-Hookean, isotropic material and a nonlinear, anisotropic material. The isotropic material is generally attributed to elastin (or elastic fibers), and the anisotropic material is generally attributed to collagen fibers (Holzapfel et al., 2000). This assumption enables analysis of how the amount and integrity of elastic and collagen fibers in the aorta change with age, vessel, and genotype from the fitted material parameters. Age-related fluctuations in c, the material constant for the isotropic, Neo-Hookean component associated with elastic fibers, were observed in ASC and DSC, with the peak occurring around 3 months of age in both genotypes. This may follow temporal patterns of elastin expression which peaks postnatally in the mouse (McLean et al., 2005; Weiss et al., 2024; Kelleher et al., 2004). Our data also showed that c depended on genotype and was lower in MU than WT. A lower value of c due to fragmented elastic fibers is consistent with the wall structure observed in MU aorta in this study and others (Cavinato et al., 2021; Dwivedi et al., 2024, 2025; Jimenez-Altayo et al., 2017; Chen et al., 2019). Previous studies have shown that the value of c correlates with elastin amounts in WT and elastin haploinsufficient ASC during maturation (Cheng et al., 2013) and in newborn elastin knockout ASC (Kim et al., 2022).

The value of $k_{2}$ and the ratio of $k_{1}∕k_{2}$ are both measures of contributions by the nonlinear, anisotropic material attributed to collagen fibers and the degree of nonlinearity of the stretch-stress curve. The higher $k_{2}$ in DSC compared to ASC indicates more nonlinear behavior and suggests a lower elastin-to-collagen ratio, which has been shown to decrease with distal location along the aorta (Liyanage et al., 2022). For both ASC and DSC, $k_{2}$ was higher in MU than WT, suggesting a lower elastin-to-collagen ratio in MU compared to WT, consistent with microstructural studies that show collagen deposition in MU ASC (Cavinato et al., 2021; Dwivedi et al., 2024, 2025) and in human aorta with MFS (Halme et al., 1985). The ratio of $k_{1}∕k_{2}$ was consistently lower for MU than WT at higher ages, indicating more nonlinear stretch-stress behavior and increased collagen contributions. Similar increases in nonlinearity of the aortic stretch-stress curve have been observed in other mouse models where increased collagen deposition may occur in response to altered elastic fiber structure and mechanical contributions (Le et al., 2015; Dwivedi et al., 2024; Cheng et al., 2013; Kim et al., 2022).

### 4.2. Physiologic biomechanical metrics

#### 4.2.1. Individual effects of age

The individual effects of age indicate significant changes induced by postnatal maturation in the mouse. Specifically, the unloaded diameters, loaded inner diameter, axial stretch ratio, stored strain energy (total, iso- and aniso-contributions), circumferential and axial stresses (total, iso- and aniso-contributions), and circumferential and axial moduli all increased between 1 and 4 months of age in ASC and DSC for WT and MU mice. Dwivedi et al. (2024) found age-dependent increases in body weight and systolic and diastolic blood pressure for WT and MU mice over the same time period suggesting overall growth of the mouse and increases in hemodynamic loads with maturation. Increases in hemodynamic loads increase the wall stresses and encourage remodeling of the aortic wall (Wagenseil et al., 2009). The deposition of elastin and collagen during this time period serves to stiffen and strengthen the aortic wall for optimal cardiovascular function (Murtada et al., 2021). Many of the physiologic biomechanical metrics peaked around 3 months of age, when the mouse is considered mature, and hemodynamic changes and ECM expression are minimal (Murtada et al., 2021; Weiss et al., 2024; Wagenseil et al., 2009; Le et al., 2011).

#### 4.2.2. Individual effects of vessel

The individual effects of vessel reveal significant differences due to location along the aortic length. Our results show that compared to DSC, ASC had higher unloaded diameters, loaded inner diameter, circumferential and axial stretch ratios, stored strain energy (total, iso- and aniso-contributions), circumferential stresses (total and aniso-contributions), axial stresses (total, iso- and aniso-contributions), and circumferential and axial moduli in WT and MU mice. These results are consistent with those reported by Bellini et al. (2016) comparing ASC and DSC and Dwivedi et al. (2025) comparing multiple aortic regions in WT and MU mice at a single time point. As the first branch of the aorta, the ASC receives the entire left ventricular cardiac output, which is decreased by the time it reaches the DSC. Walther et al. (1986) found that about 64 % of the left ventricular cardiac output is received by the DSC. The proximity to the left ventricle also means that the ASC experiences higher pulsatile flow and pressure compared to the downstream DSC (O’Rourke et al., 1980). The higher hemodynamic loads on the ASC are consistent with high physiologic biomechanical metrics. The distinct microstructural compositions of the ASC and DSC are related to their different physiological functions. The elastin-to-collagen ratio is higher in ASC than in DSC (Grant, 1967), which suggests that elasticity and stored strain energy are more important in the ASC than the DSC to reduce the work load on the heart and dampen the pulsatile pressure and blood flow for downstream tissues (Mitchell, 2018). Assadi et al. (2024) concluded that the ASC acts like a reservoir, whereas the DSC acts like conduit. The differences in physiological function might explain the alterations in physiologic biomechanical metrics based on aortic location.

#### 4.2.3. Individual effects of genotype

The individual effects of genotype indicate different biomechanical behaviors between MU and WT aorta that might relate to TAA progression in MFS. Our results show that compared to WT, MU aorta across all ages had increased unloaded diameters, increased loaded inner diameter, increased circumferential stretch ratio, decreased axial stretch ratio, decreased strain energy storage (total, iso-, and aniso-contributions), increased total and aniso-contributions of the circumferential stresses, decreased iso-contributions of the circumferential and axial stresses, and increased circumferential and axial moduli.

The increased unloaded diameters demonstrate remodeling of the aortic wall in TAA beyond just increased circumferential stretch on the wall to increase the loaded diameter. The decrease in axial stretch ratio is a common attribute of aorta with reduced (Wagenseil et al., 2005) or fragmented elastic fibers (Bellini et al., 2016; Cavinato et al., 2021; Dwivedi et al., 2024) and may serve to offload the aorta to reduce overall stiffness (Humphrey et al., 2009). Decreased strain energy storage is also a common attribute of aorta with fragmented elastic fibers (Le et al., 2015; Bellini et al., 2016; Cavinato et al., 2021) and highlights the importance of elastic fibers for this biomechanical function. Increased total circumferential stress is negatively correlated with the number of cycles before material failure in the aorta (Polzer et al., 2023), a sign of mechanical fatigue as a result of cyclic loading in the cardiac cycle that may be exacerbated with TAA. The shift in stress contributions from the isotropic material (i.e. elastic fibers) to the anisotropic material (i.e. collagen fibers) has been demonstrated in elastin knockout ASC (Kim et al., 2022), elastin haploinsufficient ASC (Cheng et al., 2013), and ASC with fragmented elastic fibers (Le et al., 2015) and highlights the dynamic remodeling process in the aorta and the compensation by collagen deposition when there are defects in elastic fibers. As elastic fibers are typically formed during late prenatal development and postnatal maturation with little deposition and assembly in adulthood, defects or reduced amounts of elastic fibers must be compensated for by other ECM proteins, such as collagen (Wagenseil et al., 2009).

ECM remodeling and the shift in stress contributions from elastic to collagen fibers is likely the cause of the increased circumferential moduli observed in MU compared to WT aorta. Bellini et al. (2016) and Dwivedi et al. (2025) found increased ASC and DSC circumferential material stiffness in two or four months old male MU mice compared to WT, respectively. Dwivedi et al. (2024) reported progressive increases in in vivo and ex vivo measures of circumferential structural and material stiffness and the ex vivo modulus at high stretch ratio, a marker for increased nonlinearity of the stretch-stress curve, for 1–4 month old male and female MU ASC compared to WT. Gharraee et al. (2022) found an increase in the ASC ex vivo circumferential modulus at high stretch ratio for a milder mouse model of MFS. ECM remodeling and increased circumferential stiffness have also been reported for human MFS aorta. Although material stiffness is challenging to measure in humans, structural stiffness can be easily measured by pulse wave velocity. Aortic pulse wave velocity was increased in adults with MFS compared to control (Jeremy et al., 1994; Westenberg et al., 2011; Salvi et al., 2018). Increased structural stiffness and pulse wave velocity alter the behavior of reflected pressure waves within the aorta, augmenting systolic instead of diastolic pressure, which increases pulse pressure, and may exacerbate dilation in MFS (Marque et al., 2001).

#### 4.2.4. Effects of interactions between independent variables

A summary of the significant effects and percent variation attributed to each variable and their interactions can be found in Supplemental Table S1. An interaction between age and genotype suggests different growth and remodeling timelines for MU and WT aorta. Every measured metric, except circumferential stretch ratio, had a significant interaction between age and genotype. Discussion of the metrics altered individually by genotype can be found above. The interaction suggests that not only are the metrics affected by genotype, but the normal rate of change of these metrics over 1–4 months of age in the mouse interacts with genotype. Interestingly, circumferential stretch was increased in MU compared to WT but then was maintained at this value across all ages with no interaction between genotype and age or individual effect of age. This may be related to an optimal stretch or deformation for smooth muscle cells to provide maximum contractility (Hill, 1938) that is dependent on the surrounding environment (i.e. ECM).

An interaction between age and vessel suggests different remodeling timelines between ASC and DSC in both genotypes. The only metrics that depended on interactions between age and vessel were unloaded diameters, loaded inner diameter, and circumferential stretch ratio. These results indicate that increases in the diameter and the age at which it reaches final adult values vary with axial location along the aorta. This may be partly related to differences in elastin and collagen expression (McLean et al., 2005; Weiss et al., 2024; Kelleher et al., 2004) and remodeling (Bendeck et al., 1991, 1994) between the two aortic segments that affect both absolute diameter and circumferential stretch ratio. Additionally, different smooth muscle cell embryonic lineages between ASC and DSC might predispose them to different developmental timelines during maturation (Pfaltzgraff et al., 2014). Because of the ASC’s proximity to the left ventricle and receiving almost the entire cardiac output, the increase in unloaded and loaded diameters is faster than the DSC (Redheuil et al., 2011).

An interaction between genotype and vessel suggests that MU ASC and DSC respond differently to the primary insult, namely reduced amounts of fibrillin-1. The unloaded diameters, deformed inner diameter, axial stretch ratio, strain energy (total, iso-, and aniso-contributions), axial stress (total and iso-contributions), and circumferential stiffness had significant interactions between genotype and vessel. These represent biomechanical metrics that may be related to the progression of the aneurysm specifically in MU ASC compared to MU DSC. The changes in diameter are characteristic of aneurysm formation, while the axial stretch ratio, stored strain energy, and circumferential stiffness are metrics that have been shown to correlate with elastic fiber fragmentation and collagen fiber deposition in mouse models of MFS (Bellini et al., 2016; Cavinato et al., 2021; Dwivedi et al., 2024, 2025; Gharraee et al., 2022; Chen et al., 2019). Clinical evidence shows that the development of TAA in different aortic segments is not concurrent (Coady et al., 1999). Patients with surgical repair of an ASC dissection are likely to get a DSC dissection later on (Charlton-Ouw et al., 2018). Therefore, site-specific monitoring is needed for better TAA treatment outcomes.

A combined effect of age, vessel, and genotype suggests complicated interactions of all three independent variables. The unloaded diameters, loaded inner diameter, axial stretch ratio, axial stress contributed by the anisotropic material, and circumferential and axial moduli were affected by the interaction of all three variables. For the loaded and unloaded diameters, a progressive increase was seen in ASC MU with age, whereas the dilation for DSC MU was milder. The increase in the unloaded outer and inner diameters indicates permanent deformation in the circumferential direction, which might be a result of ECM remodeling (Dwivedi et al., 2024). The changes in circumferential modulus for ASC were similar to the dilation rate, with large changes with age in MU ASC and small changes with age in MU DSC, but overall larger values in MU compared to WT. Sharma et al. (2009) found that loss of distensibility, a measure of structural stiffening, precedes dilation for human patients with TAA associated with Turner’s syndrome. Dilation of the aorta might be a compensatory response to a stiffer, less compliant vessel that is less able to store blood during systole and push it downstream during diastole (Lyle et al., 2017). Correlations between dilation and circumferential stiffness are consistent with our data for MU mice in this study and previous studies (Bellini et al., 2016, 2017; Dwivedi et al., 2024, 2025). Complex dependence of aortic stiffness, as measured by pulse wave velocity, on age, vessel, and MFS diagnosis has also been observed in humans (Jeremy et al., 1994; Westenberg et al., 2011; Salvi et al., 2018; Rogers et al., 2001).

### 4.3. Mixed model suggests additional biomarkers for MFS treatment

A biomarker is a quantity that can be measured clinically to determine disease progression and/or appropriate timing for intervention. Maximum diameter and/or rate of change of diameter is the current biomarker used for determining surgical intervention in TAA. However, it is not sufficient, as 60 % of patients experience dissection and/or rupture before the criterion is met (Pape et al., 2007). Additional and different types of biomarkers, such as biomechanical metrics, need to be examined for improving TAA treatment (Bazzi et al., 2022). As we do not have disease outcomes (i.e. rupture, dissection, or lifespan) as a metric in this study and we know that aortic diameter is one measure of TAA progression, we examined correlations between various biomechanical metrics and physiologic loaded inner diameter in WT and MU aorta using a multivariable mixed model.

For WT aorta, the metrics that best predicted the loaded inner diameter were the total strain energy and the circumferential stress contributed by the isotropic material, where the total strain energy contributed more to the adjusted R^2^ and positively contributed to the dilation. This suggests that energy storage ability is important during the growth and maturation of a normal, non-diseased aorta and is consistent with its physiologic function as a Windkessel (Wagenseil et al., 2009). The other predictive metric, circumferential stress contributed by the isotropic material, had a negative coefficient, which might relate to the fact that elastin expression peaks at 1 month of age and plateaus, even as the aorta keeps growing (Weiss et al., 2024).

For MU aorta, the metrics that best predicted the loaded inner diameter were the circumferential and axial moduli, with the circumferential modulus being the primary contributor. The results for MU aorta highlight the relationship between increased circumferential modulus (material stiffness) and dilation that has been shown in previous studies on mouse models of MFS (Bellini et al., 2016; Cavinato et al., 2021; Dwivedi et al., 2024, 2025; Gharraee et al., 2022; Chen et al., 2019). Correlations between increased circumferential stiffness and aortic diameter have also been found in humans with MFS (Sulejmani et al., 2017; Boczar et al., 2019; Schierling et al., 2022). The MU mixed model suggests that axial modulus plays a small, but significant role in predicting the deformed diameter. The axial modulus had a negative coefficient, demonstrating that reduced axial modulus correlated with diameter increases in MU aorta, which was the opposite of the circumferential modulus-diameter relationship. The reduction in axial modulus may be related to the reduction in axial stretch to offload the aorta and mitigate the large increases in circumferential modulus (Humphrey et al., 2009).

Measures of circumferential modulus that can be determined noninvasively in the clinic include pulse wave velocity. Boczar et al. (2019) found correlations between TAA diameter and pulse wave velocity. Schierling et al. (2022) proposed that pulse wave velocity might be used for risk stratification in TAA. Such clinical evidence, along with our findings, suggest that pulse wave velocity and arterial stiffening may be useful biomarkers for TAA management in MFS. While pulse wave velocity only measures circumferential stiffness, axial stiffness can be estimated noninvasively with patient-specific imaging and computational modeling (Parikh et al., 2024).

For both WT and MU aorta, correlations between the predicted diameter and actual diameter were moderate (R^2^ = 0.5–0.6), indicating that additional metrics are needed in the mixed model for better predictions. Dwivedi et al. (2025) showed that a mixed model including biomechanical metrics, microstructural characterization of the wall, and unloaded dimensions had strong (R^2^ = 0.88) predictive power for the loaded inner diameter for multiple aortic regions from WT and MU mice at 4 months of age. Machine learning shows promise for even more sophisticated modeling techniques. Deep learning models have been used to predict constitutive model constants (i.e. stress-stretch relationships) based on microstructural information from human aneurysm samples (R^2^ = 0.97) (Holzapfel et al., 2021) and the evolving mechanical behavior of the mouse DSC during maturation and aging from microstructural characterization and biaxial mechanical data (R^2^ = 0.92) (Linka et al., 2022).

### 4.4. Limitations and future work

We investigated TAA progression in a mouse model of MFS. The findings should be cautiously extended to human patients to test their sensitivity and applicability. We combined the results for males and females due to low statistical power for the expected effect of sex, but sexual dimorphism should be further evaluated. TAA incidence, progression, and survival is sex dependent in MU mice (Dwivedi et al., 2024, 2025; Zaradzki et al., 2022; Chen et al., 2017; Budbazar et al., 2023). About 50 % of MU male mice and 70 % of MU female mice survived to 4 months of age in our study (Dwivedi et al., 2024), consistent with previous work (Zaradzki et al., 2022; Chen et al., 2017; Budbazar et al., 2023), hence our data are biased towards surviving mice. Active mechanical testing that includes contributions of smooth muscle cells (Cantalupo et al., 2025), and simulation of the hemodynamics and wall shear stresses (Bazzi et al., 2022), should also be studied to complement our passive biaxial testing results. Other biophysical phenomena, such as fluid and solute transport across the aortic wall, may also play a role in TAA progression (Crandall et al., 2023) and should be investigated as possible biomarkers. Multiphoton imaging was analyzed qualitatively, but in-depth analysis of elastic fiber integrity and collagen fiber content has been shown to correlate with TAA growth and should be further investigated as a possible biomarker and/or contributing factor in a predictive model (Cavinato et al., 2021; Dwivedi et al., 2024, 2025; Sulejmani et al., 2017). The remaining segments of the aorta, including the superior and inferior abdominal aorta, should also be evaluated mechanically to better understand regional heterogeneity in aneurysm formation associated with MFS (Dwivedi et al., 2025; Takayama et al., 2009). We showed that total stored strain energy and circumferential modulus were the greatest contributors to a mixed model for inner diameter predictions in WT and MU aorta, respectively, indicating that each biomarker may be a useful predictor of growth in WT aorta and TAA progression in MU aorta. We did not investigate mechanisms to explain why these biomarkers correlate with aortic diameter. Future work is needed to determine the existence and extent of any causal relationships.

## Supplementary Material

Supp data

Supplementary data to this article can be found online at https://doi.org/10.1016/j.jmbbm.2025.107105.

## Figures and Tables

**Table 1:** Average mean blood pressure for WT and MU mice at ages 1, 2, 3, and 4 months (Dwivedi et al., 2024).

Genotype	WT	MU
	
Age (mo)	Mean blood pressure (mmHg)	
1	99 ± 9	96 ± 5
2	106 ± 8	109 ± 10
3	104 ± 5	105 ± 7
4	106 ± 5	104 ± 7

**Table 2:** Mixed model results for predicting the deformed inner aortic diameter from biomechanical metrics for WT and MU.

Vessel	WT	MU
**R^2^**	0.58	0.53
**p-value**	$W_{\text{tot}}<0.00001$; $\sigma_{\theta ,\text{iso}}<0.00001$	$\zeta_{\theta }<0.00001$; $\zeta_{z}<0.00001$
**VIF**	2.35	1.94
**Coefficients**	$W_{\text{tot}}=7.55$; $\sigma_{\theta ,\text{iso}}=-3.70$	$\zeta_{\theta }=0.46$; $\zeta_{z}=-0.60$

## Data Availability

Data will be made available on request.
