# Supplementary material for: Different physiologic biomechanical metrics correlate with aortic diameter increases in normal maturation compared to aneurysm progression in mice
Source: J Mech Behav Biomed Mater. Author manuscript; Available in PMC 2025 Sep 5. (PMC12412189; doi:10.1016/j.jmbbm.2025.107105)
Supplement: Supp data [file NIHMS2105954-supplement-Supp_data.pdf]

Supplemental Figures and Tables

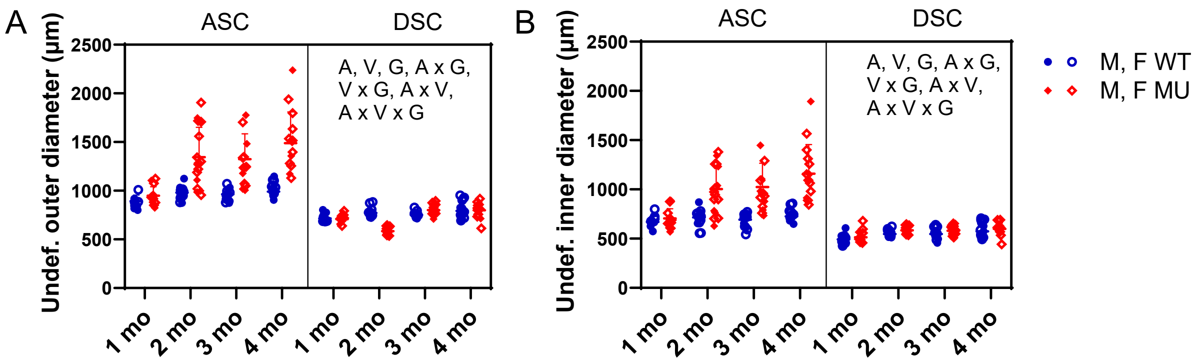

Supplemental Figure S1. Undeformed outer diameter (A) and undeformed inner diameter (B) of for each group. Letters indicate significant effects by 3-way ANOVA for the independent variables age (A), vessel (V), and genotype (G) and all interactions. Males and females are plotted with different symbols but are not separated by sex for statistical analyses. Individual data points and mean ± SD are shown. N = 10 – 19/group.

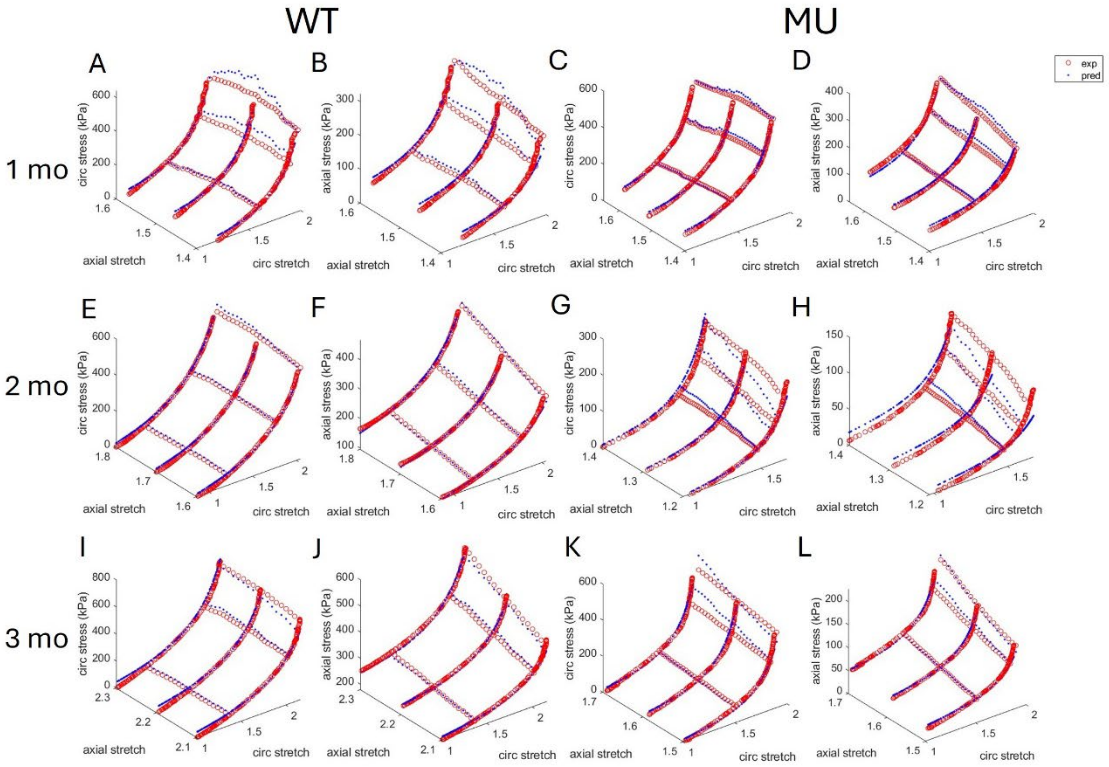

Supplemental Figure S2. Representative experimental (exp) and HGO-model predicted (pred) stretch-stress data for ASC WT (A, B, E, F, I, J) and MU (C, D, G, H, K, L) at age = 1 (A - D), 2 (E - H), and 3 months (I – L).

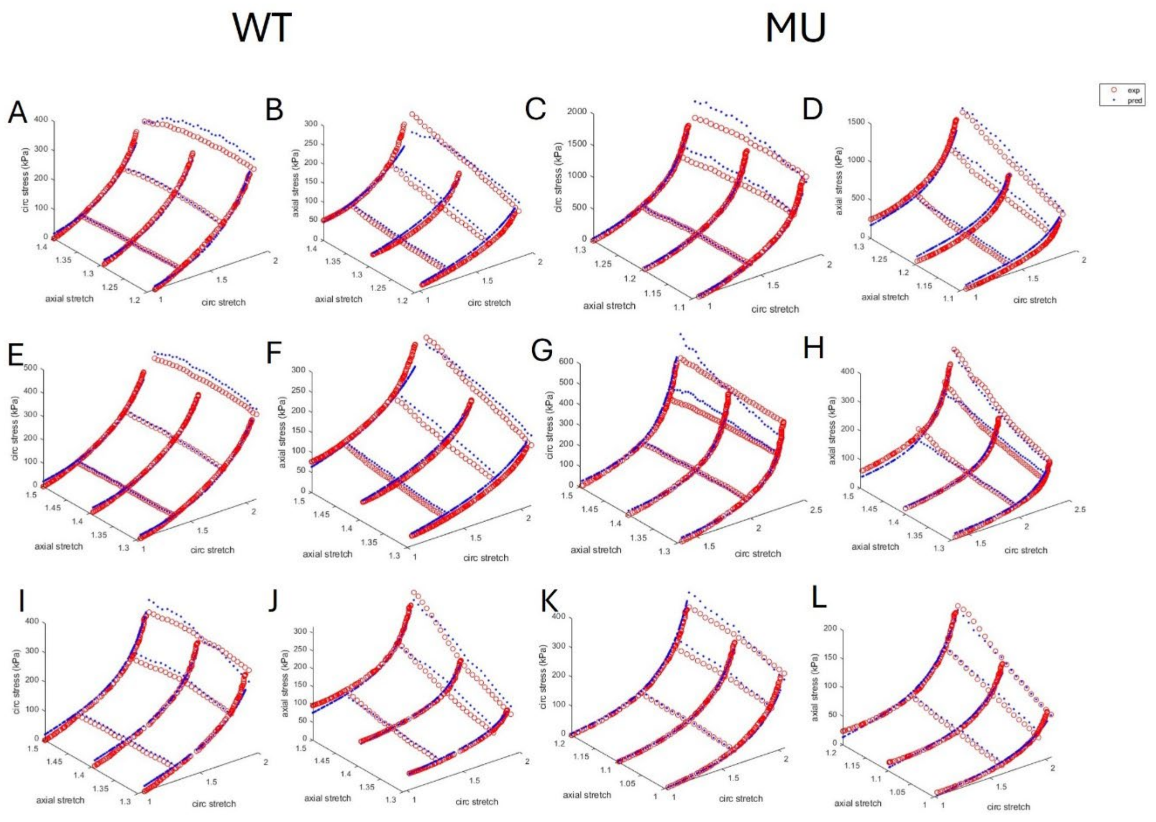

Supplemental Figure S3. Representative experimental (exp) and HGO-model predicted (pred) stretch-stress data for DSC WT (A, B, E, F, I, J) and MU (C, D, G, H, K, L) at age = 1 (A - D), 2 (E - H), and 3 months (I – L).

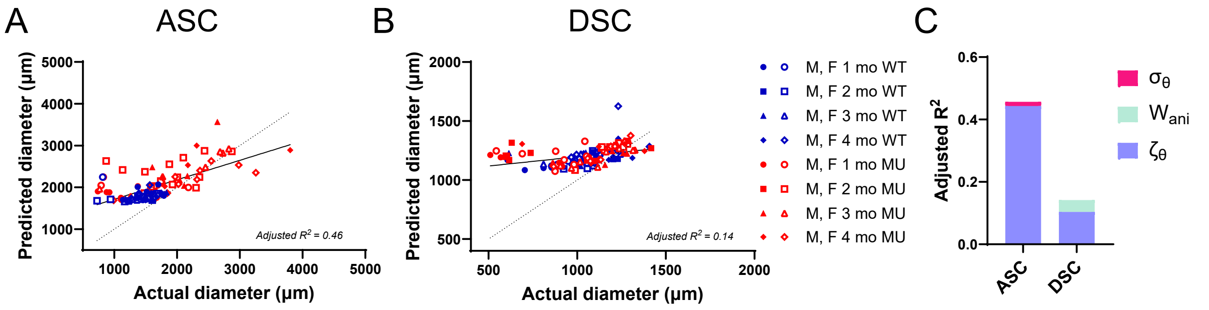

Supplemental Figure S4. The best mixed model results predicting the deformed inner diameter for ASC (A) and DSC (B) from the physiologic biomechanical parameters (C). σ_θ = total circumferential stress; W_{ani} = strain energy contributed by the anisotropic, nonlinear material, ζ_θ = circumferential modulus. The best fit line (solid), identity line (dotted), and adjusted R^2 are shown in panels A and B. The relative contributions of each biomechanical parameter to the adjusted R^2 are shown in panel C. The p-value, coefficients, and VIF are provided in Supplemental Table S1.

Supplemental Table S1. Summary of % total variation from three-way ANOVA to determine the contributions of age, vessel and genotype and their interactions to the measured parameters. Colored text represents significant p value <0.05. The associated manuscript figure is listed in the last column. d_i= deformed inner diameter; λ_θ = mid wall circumferential stretch ratio; λ_z = mid wall axial stretch ratio; R^2= goodness of fit for the constitutive model to the experimental data; c is an elastic modulus-like parameter for the isotropic, Neo-Hookean material; k_1 is an elastic modulus-like parameter for the anisotropic, nonlinear material; k_2 is a dimensionless parameter associated with nonlinearity for the anisotropic, nonlinear material; α = angle of the two symmetric fiber families with respect to the circumferential direction; W_{total} = total strain energy; W_{iso} = strain energy contributed by the isotropic, Neo-Hookean material; W_{aniso} = strain energy contributed by the anisotropic, nonlinear material; $\sigma_{\theta,total}$ = total circumferential stress; $\sigma_{\theta,iso}$ = circumferential stress contributed by the isotropic, Neo-Hookean material; $\sigma_{\theta,aniso}$ = circumferential stress contributed by the anisotropic, nonlinear material; $\sigma_{z,total}$ = total axial stress; $\sigma_{z,iso}$ = axial stress contributed by the isotropic, Neo-Hookean material; $\sigma_{z,aniso}$ = axial stress contributed by the anisotropic, nonlinear material; ζ_θ= circumferential incremental modulus; ζ_z= axial incremental modulus; D_o= undeformed outer diameter; D_i= undeformed inner diameter.

Parameter	A	V	G	A × V	A × G	V × G	A × V × G	Figure
d_i	9.24	36.30	4.55	1.44	2.20	4.26	1.82	1A
λ_θ	1.82	1.76	2.86	3.62	0.68	0.06	2.85	1B
λ_z	3.17	37.20	6.69	0.75	4.66	1.51	2.69	1C
R^2	1.01	5.34	13.39	5.80	2.07	2.44	0.70	3A
c	8.32	0.36	4.02	0.90	4.61	0.13	2.59	3B
k_1	3.33	1.82	6.90	4.39	4.07	0.66	0.69	3C
k_2	3.26	12.68	10.87	2.60	0.03	0.26	0.55	3D
k_1/k_2	3.00	4.24	13.54	1.39	1.31	5.29	0.20	3E
α	2.52	33.43	0.04	0.70	2.95	1.60	0.06	3F
W_{total}	3.11	20.71	6.50	0.84	4.82	3.71	2.11	4A
W_{iso}	5.67	7.83	5.24	0.41	5.74	2.32	2.08	4B
W_{ani}	2.69	30.15	4.47	1.37	3.50	3.50	1.29	4C
$\sigma_{\theta,total}$	4.41	24.48	1.37	0.88	3.53	0.05	2.11	5A
$\sigma_{\theta,iso}$	6.59	0.42	2.49	0.31	5.14	1.07	2.26	5B
$\sigma_{\theta,ani}$	3.66	30.17	4.08	1.19	3.73	0.04	1.17	5C
$\sigma_{z,total}$	5.11	20.64	2.50	1.47	5.94	2.50	2.69	5D
$\sigma_{z,iso}$	7.01	8.14	7.70	0.36	6.72	1.48	1.96	5E
$\sigma_{z,ani}$	3.35	23.42	0.19	2.19	4.50	2.33	2.32	5F
ζ_θ	3.90	13.7	20.51	0.81	3.73	2.36	1.94	6A
ζ_z	6.70	6.22	2.31	0.86	5.48	1.31	3.12	6B
D_o	6.36	44.39	6.38	3.31	2.52	9.82	2.74	S1A
D_i	7.02	39.13	11.15	1.78	2.54	7.20	2.38	S1B

Supplemental Table S2. Mixed model results for predicting deformed inner aortic diameter from biomechanical metrics for ASC and DSC.

**Vessel**	**ASC**	**DSC**
**R^2**	0.46	0.14
**p-value**	$\sigma_{\theta, total}$ = 0.0308; ζ_{θ} < 0.0001	W_{ani} = 0.0079; ζ_{θ} = 0.0147
**VIF**	2.14	1.17
**Coefficients**	$\sigma_{\theta, total}$ = -1.10; ζ_{θ} = 0.33	W_{ani} = 4.44; ζ_{θ} = 0.07
